# Doppler Versus Thermodilution-Derived Coronary Microvascular Resistance to Predict Coronary Microvascular Dysfunction in Patients With Acute Myocardial Infarction or Stable Angina Pectoris

**DOI:** 10.1016/j.amjcard.2017.09.012

**Published:** 2018-01-01

**Authors:** Rupert P. Williams, Guus A. de Waard, Kalpa De Silva, Matthew Lumley, Kaleab Asrress, Satpal Arri, Howard Ellis, Awais Mir, Brian Clapp, Amedeo Chiribiri, Sven Plein, Paul F. Teunissen, Maurits R. Hollander, Michael Marber, Simon Redwood, Niels van Royen, Divaka Perera

**Affiliations:** a British Heart Foundation Centre of Excellence and National Institute for Health Research Biomedical Research Centre, Cardiovascular Division, Rayne Institute, St Thomas' Hospital, King's College London, London, United Kingdom; b Department of Cardiology, VU University Medical Center, Amsterdam, The Netherlands; c Multidisciplinary Cardiovascular Research Centre & Division of Biomedical Imaging, Leeds Institute of Cardiovascular and Metabolic Medicine, University of Leeds, Leeds, United Kingdom

## Abstract

Coronary microvascular resistance is increasingly measured as a predictor of clinical outcomes, but there is no accepted gold-standard measurement. We compared the diagnostic accuracy of 2 invasive indices of microvascular resistance, Doppler-derived hyperemic microvascular resistance (hMR) and thermodilution-derived index of microcirculatory resistance (IMR), at predicting microvascular dysfunction. A total of 54 patients (61 ± 10 years) who underwent cardiac catheterization for stable coronary artery disease (n = 10) or acute myocardial infarction (n = 44) had simultaneous intracoronary pressure, Doppler flow velocity and thermodilution flow data acquired from 74 unobstructed vessels, at rest and during hyperemia. Three independent measurements of microvascular function were assessed, using predefined dichotomous thresholds: (1) coronary flow reserve (CFR), the average value of Doppler- and thermodilution-derived CFR; (2) cardiovascular magnetic resonance (CMR) derived myocardial perfusion reserve index; and (3) CMR-derived microvascular obstruction. hMR correlated with IMR (rho = 0.41, p <0.0001). hMR had better diagnostic accuracy than IMR to predict CFR (area under curve [AUC] 0.82 vs 0.58, p <0.001, sensitivity and specificity 77% and 77% vs 51% and 71%) and myocardial perfusion reserve index (AUC 0.85 vs 0.72, p = 0.19, sensitivity and specificity 82% and 80% vs 64% and 75%). In patients with acute myocardial infarction, the AUCs of hMR and IMR at predicting extensive microvascular obstruction were 0.83 and 0.72, respectively (p = 0.22, sensitivity and specificity 78% and 74% vs 44% and 91%). We conclude that these 2 invasive indices of coronary microvascular resistance only correlate modestly and so cannot be considered equivalent. In our study, the correlation between independent invasive and noninvasive measurements of microvascular function was better with hMR than with IMR.

Up to 50% of patients have microvascular obstruction (MVO)^1^ after primary percutaneous coronary intervention (PPCI), resulting in worse clinical outcomes.^2^ MVO reflects microvascular dysfunction (MVD) due to distal embolization of the thrombus, endothelial dysfunction, reperfusion injury, and intramyocardial hemorrhage.^3^ MVD also indicates an adverse prognosis in the setting of stable coronary artery disease.^4^ Elevated coronary microvascular resistance (MVR) is the hallmark of MVD. Two invasive indices of MVR are now described. Both derive MVR from simultaneous distal coronary artery measurements of pressure and flow during hyperemia using intra-coronary guidewires. However, the index of microcirculatory resistance (IMR)^5^ estimates flow with thermodilution, whereas hyperemic microvascular resistance (hMR) incorporates Doppler flow velocity.^6^ Both indices have separately been shown to predict infarct size,^7^, ^8^ MVO,^8^ regional wall motion,^7^ and adverse left ventricular (LV) remodeling.^7^ However, to date, no study has compared hMR and IMR against invasive and noninvasive measurements of MVD in humans. Our study aims were to determine the level of agreement between IMR and hMR across a range of MVR and to compare the ability of IMR and hMR to predict independent invasive and noninvasive measurements of MVD.

## Methods

In this prospective, 2-center study, patients who underwent coronary angiography were enrolled at St Thomas Hospital, London, United Kingdom, and at the VU University Medical Center, Amsterdam, The Netherlands. To sample a wide range of MVR, we enrolled 2 groups: those with stable angina and those presenting with an acute myocardial infarction (AMI), defined as a cardiac biomarker elevation in association with characteristic electrocardiographic changes and/or typical symptoms. In patients with AMI, measurements were made in the infarct artery after percutaneous coronary intervention and in an angiographically normal reference artery when feasible. Exclusion criteria were hemodynamic instability or cardiogenic shock, significant LV dysfunction, previous coronary artery bypass grafting, severe co-morbidity, left main stem disease, and standard contraindications to cardiovascular magnetic resonance (CMR). The protocols were approved by the NRES London Westminster Medical Ethics Review Committee and the Institutional Review Board of VU University Medical Center in Amsterdam. All patients were asked to give a written informed consent.

**Figure 1:**
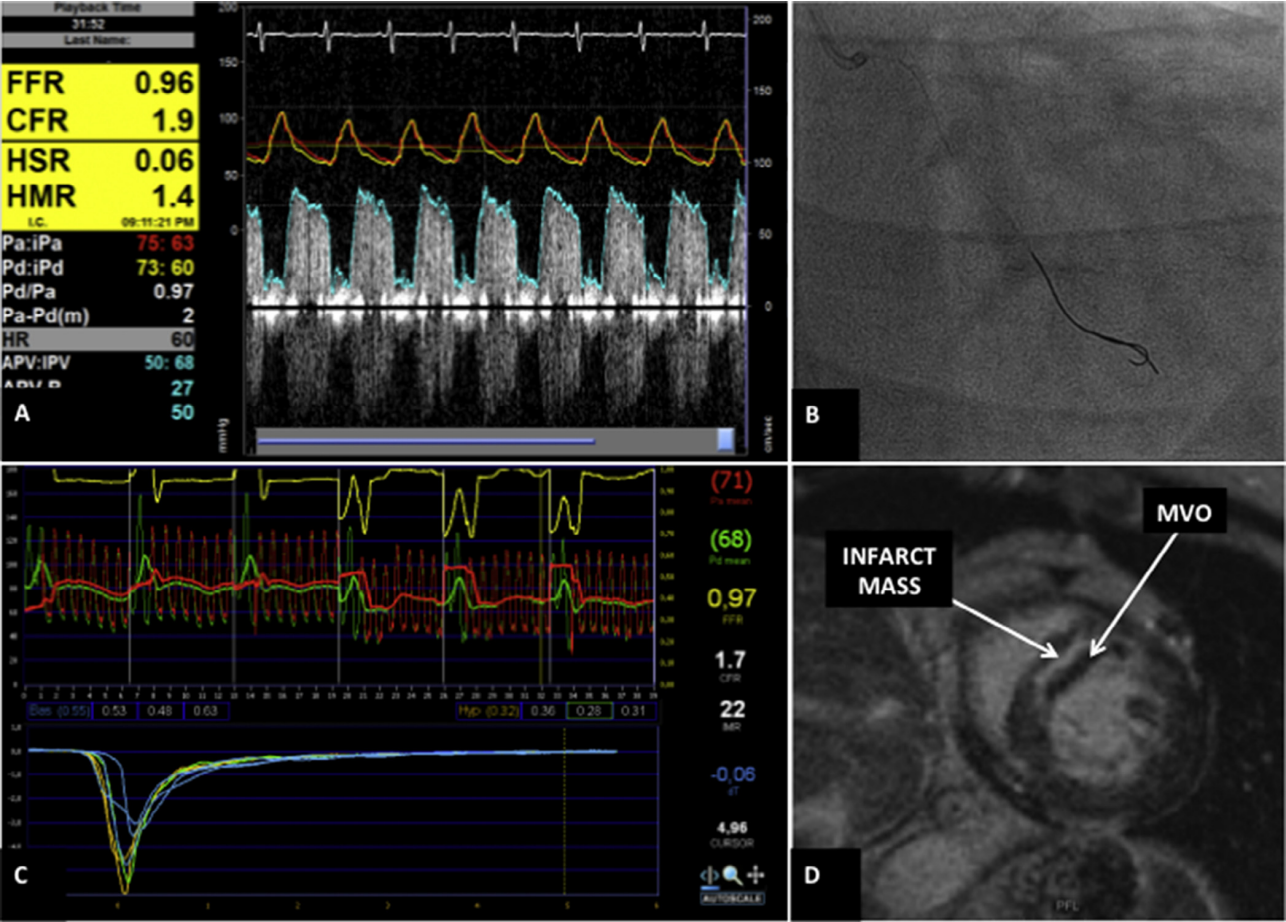
Cardiac catheterization protocol used to derive invasive measurements of microvascular resistance. *(A)* Combomap Console (Volcano Corporation, San Diego, California) displaying continuous aortic and P_d_ and Doppler flow velocity. *(B)* Coronary angiographic image demonstrating a 0.014-inch ComboWire (Volcano Corporation) and a 0.014-inch pressure wire (St Jude Medical, Uppsala, Sweden) placed in equivalent positions in the distal circumflex artery. *(C)* St Jude Console (St Jude Medical) displaying aortic and P_d_, and 3 T_mn_ measurements at both baseline and during steady-state hyperemia. *(D)* Late gadolinium enhancement cardiac magnetic resonance image 5 days after a revascularized acute ST-segment elevation myocardial infarction of the left anterior descending coronary artery. This short-axis view shows a hypoenhanced core of MVO within a hyperenhanced area of infarcted tissue in the anteroseptal myocardium. hMR = hyperemic microvascular resistance; hSR = hyperemic stenotic resistance; iPa = instantaneous aortic pressure; iPd = instantaneous distal coronary artery pressure.

Measurements were taken in coronary arteries without hemodynamically significant coronary artery disease (defined as fractional flow reserve >0.80), or immediately after a successful percutaneous coronary intervention in patients with a significant coronary artery stenosis. After calibrating and normalizing to aortic root pressure through a 6-F guiding catheter, a 0.014-inch dual pressure and Doppler flow velocity tipped sensor guidewire (ComboWire Guidewire; Philips Volcano, San Diego, California) and a 0.014-inch pressure wire (with temperature thermistors on the distal shaft and tip; St Jude Medical, St. Paul, Minnesota) were advanced to the distal vessel (>5 cm from the coronary ostia). The pressure transducers of each wire were positioned adjacent to each other (Figure 1). The following measurements were taken after the administration of intracoronary nitrates (200 to 300 mcg): aortic pressure (P_a_), distal coronary artery pressure (P_d_), Doppler-derived average peak velocity, and thermodilution-derived transit mean time.^5^, ^9^ Measurements were taken at rest and during peak hyperemia with intravenous adenosine (140 mcg/kg/min). The following were then calculated as previously described in all patients: fractional flow reserve,^10^ hMR,^11^ IMR,^5^ and the Doppler-^12^ and thermodilution-derived coronary flow reserve (CFR)^9^. In patients with AMI the corrected TIMI frame count was also calculated.^13^ Doppler flow velocity tracings of insufficient quality were discarded from the analysis. CFR was then calculated as the average of Doppler-derived CFR and thermodilution-derived CFR. Investigators performing data analyses were blinded to all clinical data. CMR scans were performed using either a 3-T magnetic resonance (MR) scanner (St Thomas' Hospital, London: Achieva; Philips Healthcare, Best, The Netherlands) or a 1.5-T MR scanner (VU University Medical Center, Amsterdam: Magnetom Avanto; Siemens, Erlangen, Germany). Cine images were acquired in 2-, 3-, and 4-chamber orientations and in a whole LV short-axis stack using a steady-state free precession sequence. CMR high-resolution stress (adenosine 140 mcg/kg/min for 4 minutes) and rest perfusion scans were performed exclusively on a 3-T MR scanner, within 48 hours of MVR measurements, using gadolinium contrast. In patients with AMI, late gadolinium enhancement images were obtained 15 minutes after the last CMR contrast injection. LV ejection fraction and LV mass were calculated from cine images. The myocardial perfusion reserve index (MPRI) was derived from the semiquantitative perfusion analysis, as previously described, to provide territory-specific values to match invasive data, using a 16-segment American Heart Association model (Supplementary Figure S7 online data supplement).^14^ MVO was manually delineated from late gadolinium enhancement images as an area of hypoenhancement within an infarcted LV mass (Figure 1).^15^ Extensive MVO was a predefined dichotomous variable when there was >2 ml MVO volume present.^16^ Further details on the methods can be found in the online data supplement.

Statistical analyses were performed using GraphPad Prism 6.0 (GraphPad, San Diego, California) and MedCalc Statistical Software version 12.7.8 (MedCalc Software, Ostend, Belgium). Continuous variables were tested for normality using the Shapiro-Wilk test and were presented as mean ± standard deviation when data were normally distributed or as median with interquartile range when data were non-normally distributed. Correlations between hMR and IMR, and each with CFR, MPRI, and MVO, were assessed using Spearman (rho) analyses. MVD was defined dichotomously for each independent outcome variable: CFR <2.0,^17^ MPRI <1.0,^14^, ^18^ and extensive MVO.^16^ Receiver operating characteristic analysis was performed to determine the best cut-off values for predicting MVD using each method, and comparisons were made using the DeLong method. *P* values of <0.05 were considered significant.

## Results

**Figure 2:**
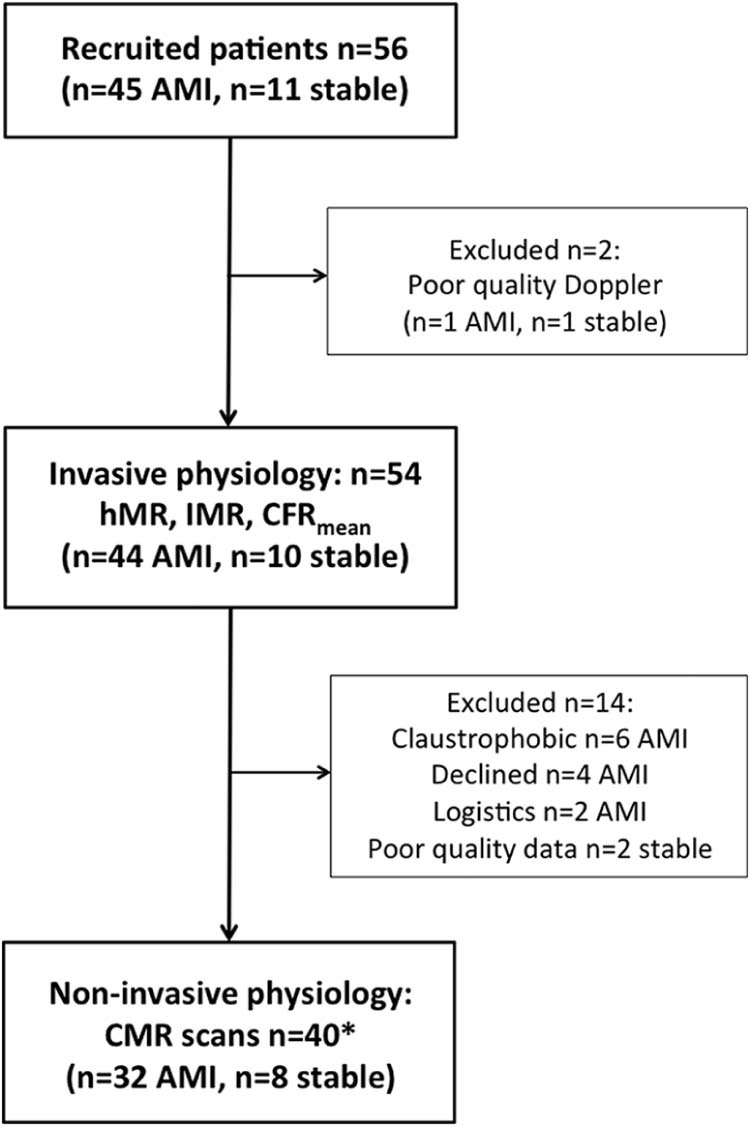
Flow of patients through the study. Two patients (4%) were excluded because of poor quality Doppler traces, leaving 54 patients (10 patients with stable angina and 44 patients with AMI: 33 with STEMI and 11 with non-STEMI) with 74 complete invasive physiology datasets (Table 1; those with a full hMR, an index of microcirculatory resistance, and a coronary flow reserve dataset from at least 1 vessel). Invasive and CMR physiologic data were acquired in 40 patients (Table 2; 8 patients with stable angina and 32 patients with AMI: 27 with STEMI and 5 with non-STEMI). Fourteen patients were excluded because of claustrophobia, patient preference (decreased), being too obese to have a CMR scan (logistics), or because of poor quality perfusion data from an inadequate breath-hold. * CMR infarct size and MVO measurements were obtained in all 32 patients with AMI (27 with STEMI and 5 with non-STEMI), whereas CMR perfusion was only performed on high-resolution 3-T perfusion scans in 23 patients (8 patients with stable angina and 15 patients with AMI).

**Figure 3:**
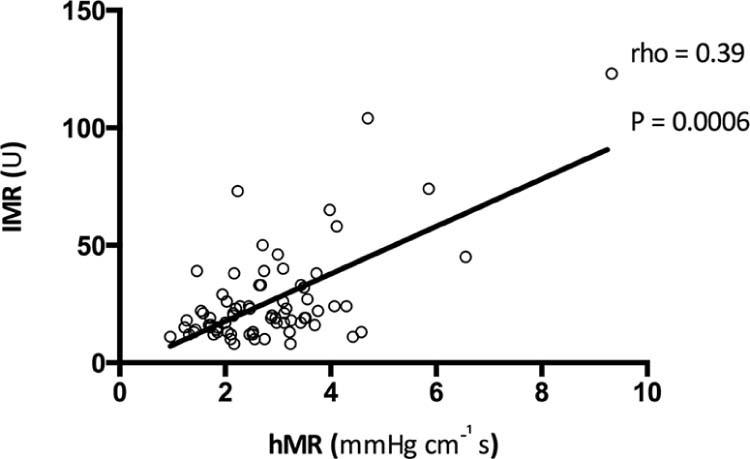
Correlation of hMR versus the IMR.

The flow of patients through the study is shown in Figure 2. Two patients (4%) were excluded because of poor quality Doppler traces, leaving 54 patients (10 patients with stable angina and 44 patients with AMI: 33 with ST-segment elevation myocardial infarction [STEMI] and 11 with non-STEMI) with 74 complete invasive physiology datasets (Table 1). Invasive and CMR physiologic data were acquired in 40 patients (8 patients with stable angina and 32 patients with AMI: 27 with STEMI and 5 with non-STEMI; Table 2). The time between invasive measurements and CMR scans was 24 hours (7 to 49 hours). In the enrolled population (see Table 1), the hMR was 2.60 (1.99 to 3.43) mm Hg·cm^-1^·s and the IMR was 19.0 (13.0 to 29.8) U. hMR significantly correlated with IMR (rho = 0.39, p = 0.0006; Figure 3). Baseline and hyperemic thermodilution transit mean time values were 0.56 (0.35 to 0.92) seconds and 0.27 (0.18 to 0.39) seconds, respectively. Baseline and hyperemic Doppler average peak velocity values were 15.3 (12.0 to 20.7) cm⋅s^-1^ and 29.4 (21.5 to 37.6) cm⋅s^-1^, respectively. Transit mean time values correlated significantly with values at baseline (rho = −0.36, p = 0.002) and hyperemia (rho = −0.41, p = 0.0003). There was a strong correlation between Doppler-derived CFR 1.90 (1.46 to 2.21) and thermodilution-derived CFR 1.82 (1.50 to 2.47) (rho = 0.61, p <0.0001).

**Table 1:** Clinical demographics and angiographic characteristics of the 54 patients

Variable	AMI Patients (n = 44)	Angina Pectoris (n = 10)
Men	40 (90)	9 (90)
Age (years)	60.2 ± 10.6	61.7 ± 9.0
Body Mass Index (kg/m^2^)	26.9 ± 3.7	29.8 ± 3.4
Hypertension	29 (64)	7 (64)
Diabetes Mellitus	21 (47)	3 (27)
Hypercholesterolemia	36 (80)	9 (82)
Smoker	30 (67)	8 (73)
Non-culprit/Non-treated Measurements		
LAD / LC / Right	9/2/7	6/3/0
Fractional Flow Reserve	0.95 ± 0.06	0.89 ± 0.04
Culprit/treated Measurements		
LAD/LC/Right	24 / 7 / 10	3 / 0 / 3
Fractional Flow Reserve (post PCI)	0.93 ± 0.06	0.92 ± 0.05
Acute Myocardial Infarction Characteristics		
Corrected TIMI frame count	17 (10–26)	n/a
Peak Troponin T, µg/L	1075 (203–7189)	n/a

Data are number (%), mean±SD or median (IQR).

LAD = left anterior descending; LC = left circumflex artery; PCI = percutaneous coronary intervention.

**Table 2:** Cardiac Magnetic Resonance (CMR) data

Variable	All patients
Duration between invasive measurements and CMR, hours	24 (7, 49)
Semi-quantitative CMR analysis (31 datasets from 23 patients*)	
Myocardial Perfusion Reserve Index	1.07 (0.86, 1.49)
Volumetric analysis (40 datasets from 40 patients)	
Left Ventricular End Diastolic Volume, ml	174 (150, 200)
Left Ventricular End Systolic Volume, ml	81 (55, 119)
Left Ventricular Ejection Fraction, %	52 (41, 63)
Microvascular Obstruction (32 datasets from 32 patients)	
Evidence of Microvascular Obstruction, number	13
Evidence of extensive† Microvascular Obstruction, number	10
Quantitative infarct size analysis (32 datasets from 32 patients)	
Infarct Size, g	22.5 (5.1, 35.2)
Infarct Size % of Left Ventricular mass	14.3 (4.5, 24.8)

Data are number, median (interquartile range) or mean ± SD.

* Includes MPRI values from corresponding culprit / non-culprit vessels.

† More than 2mls volume.

**Figure 4:**
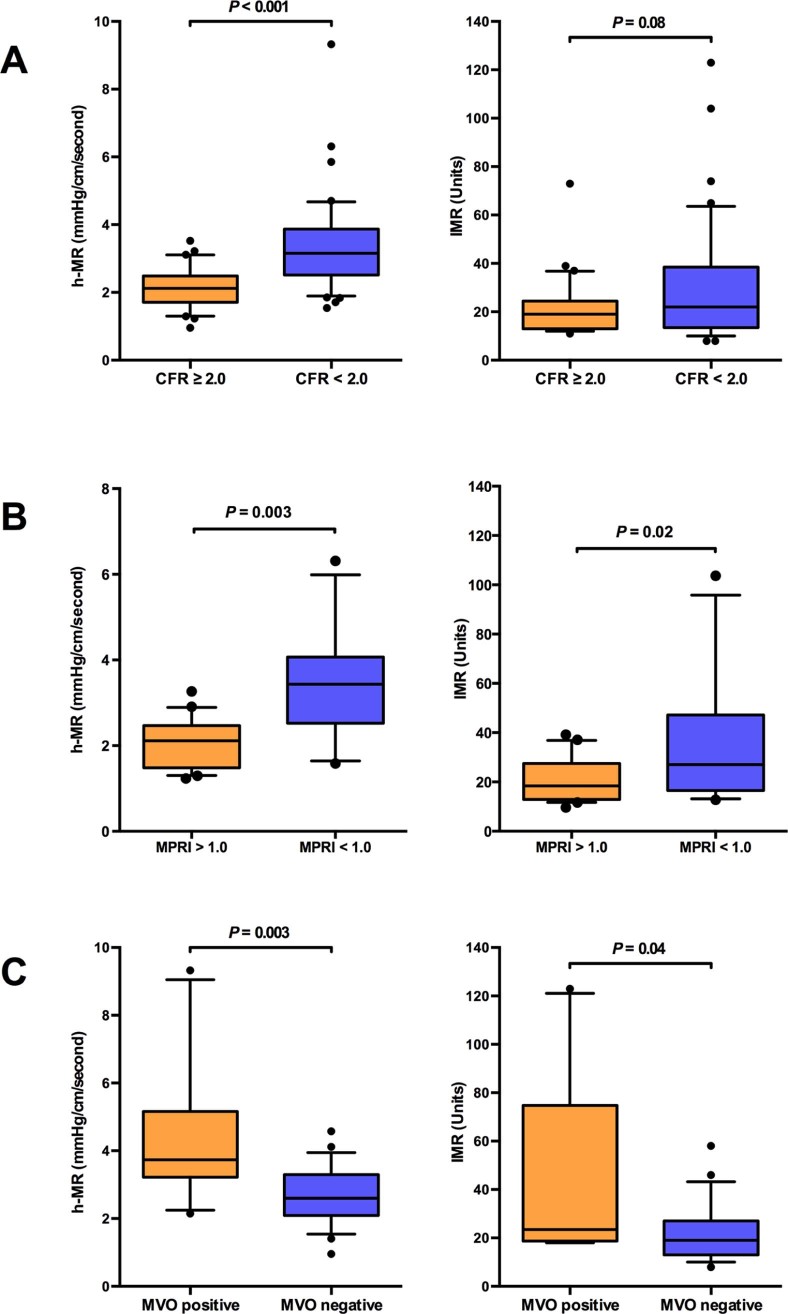
hMR and IMR invasively measured in patients with and without evidence of microvascular dysfunction as evidenced by *(A)* invasive CFR, *(B)* noninvasive myocardial perfusion reserve index, and *(C)* noninvasive extensive microvascular obstruction. Boxes represent the median and the interquartile range with whiskers as the 10th to 90th percentiles, and values outside the 10th to the 90th percentiles are presented as individual data points.

**Figure 5:**
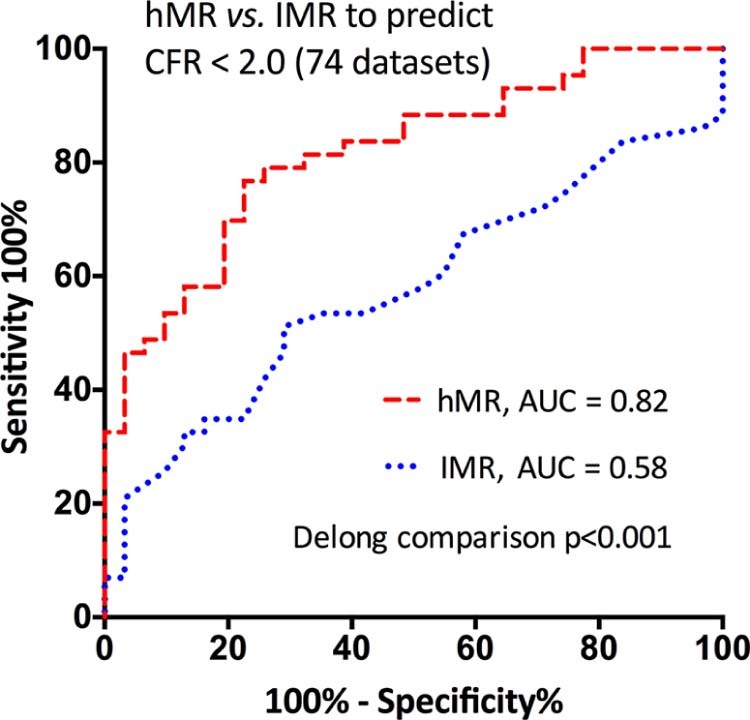
Performance of invasive indexes of microvascular resistance versus an invasive standard of coronary microvascular dysfunction: receiver operating characteristic analysis. Accuracy of hMR versus IMR in predicting a CFR of < 2.0 in vessels with a fractional flow reserve of >0.80. The optimal thresholds were ≥2.5 mm Hg·cm^-1^·s for hMR and ≥21.5 U for IMR.

hMR and IMR correlated with CFR (hMR, rho = −0.52, p <0.0001; IMR, rho = −0.24, p = 0.04). hMR values were higher in patients with MVD defined dichotomously by CFR (3.16 vs 2.12 mm Hg·cm^-1^·s, p <0.0001; Figure 4), but there was no difference between groups using IMR (22 vs 19 U, p = 0.25; Figure 4). Delong receiver-operator characteristic analysis demonstrated that hMR had superior diagnostic accuracy compared with IMR at predicting MVD: area under curve (AUC) 0.82 versus 0.58, p <0.001 (Figure 5). A threshold of  ≥ 2.5  mm Hg·cm^-1^·s for hMR provided the highest sensitivity (0.77) and specificity (0.77) for detecting MVD, whereas the optimal threshold for IMR was ≥21.5 U, with a sensitivity of 0.51 and a specificity of 0.71.

**Figure 6:**
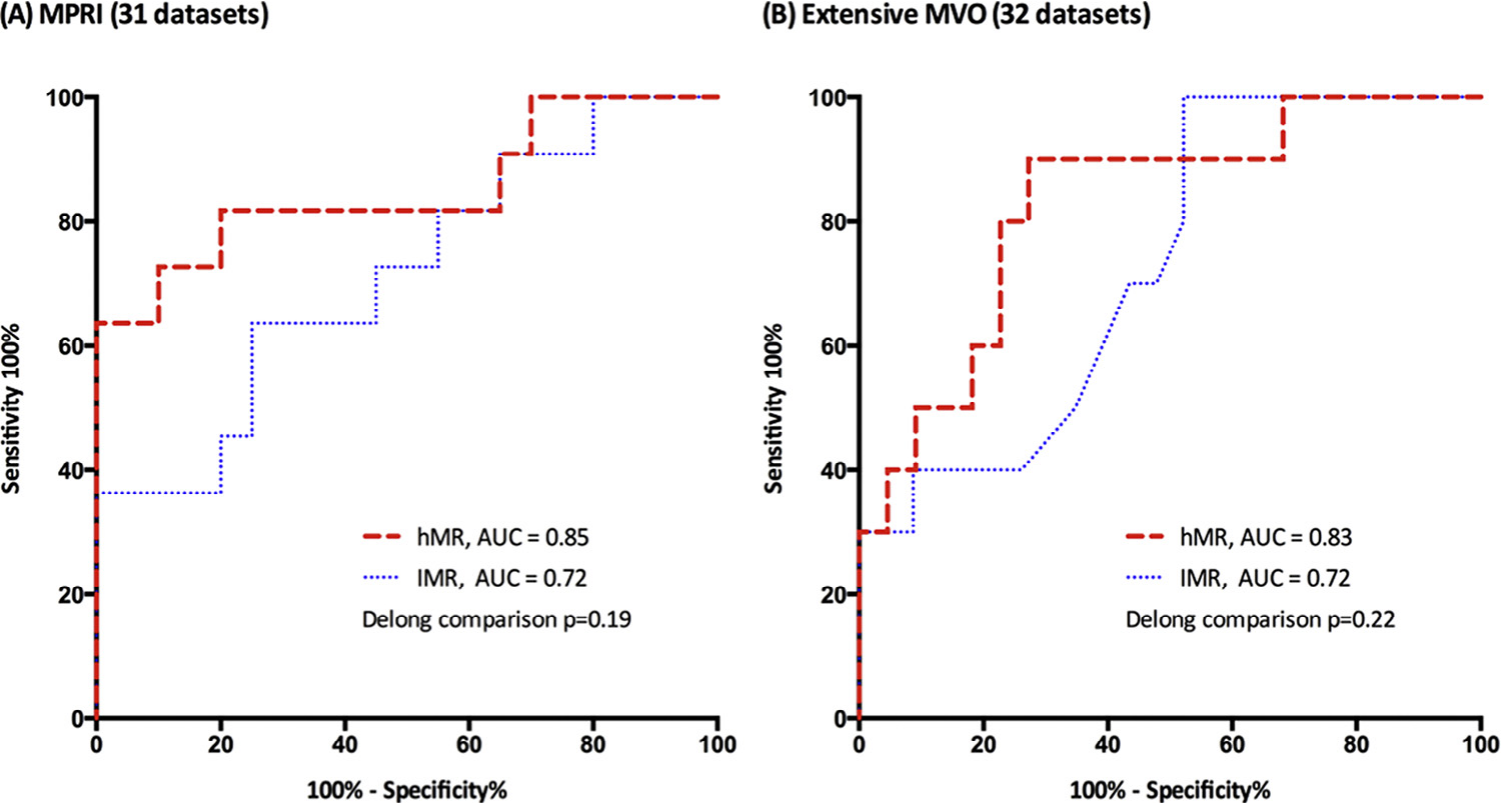
Performance of invasive indexes of microvascular resistance versus noninvasive markers of coronary microvascular dysfunction: receiver operating characteristic analysis. *(A)* Accuracy of hMR and IMR in predicting the myocardial perfusion reserve index of <1.0, a noninvasive marker of coronary microvascular dysfunction. The calculated cut-off values were ≥2.5 mm Hg·cm^-1^·s for hMR and ≥25 U for IMR. *(B)* Accuracy of hMR and IMR in predicting the presence or the absence of extensive microvascular obstruction (>2ml),^19^ a noninvasive standard of coronary microvascular dysfunction in acute myocardial infarction. The best cut-off values were ≥3.25 mm Hg·cm^-1^·s for hMR and ≥40 U for IMR.

hMR was significantly correlated with MPRI (rho = −0.58, p <0.001), but IMR was not (rho = −0.27, p = 0.15). hMR and IMR values were higher in patients with MVD, defined dichotomously by MPRI (hMR: 3.43 vs 2.11 mm Hg·cm^-1^·s, p <0.001, IMR: 27.0 vs 18.4 U, p = 0.02; Figure 4). Receiver-operator characteristic analysis showed that hMR had a numerically superior diagnostic accuracy over IMR to predict MPRI, although the difference did not reach statistical significance (AUC, 0.85 vs 0.72, p = 0.19) (Figure 6). A threshold of  ≥2.5 mm Hg·cm^-1^·s for hMR provided the optimal sensitivity (0.82) and specificity (0.80) for predicting MVD. The best cut-off value for IMR was ≥24.0 U, with poorer sensitivity (0.64) and specificity (0.75).

In the patients with AMI with invasive and CMR data (see Figure 2), MVO was visible in 42% of the patients. In these patients, the MVO volume was 3.2 ml (2.0 to 5.2). Both infarct-related artery hMR and IMR measurements correlated with MVO volume (hMR, rho = 0.46, p = 0.001; IMR, rho = 0.36, p = 0.01). hMR and IMR values were both significantly higher when there was evidence of extensive MVO (hMR 3.74 vs 2.60 mm Hg·cm^-1^·s, p = 0.003; IMR 23.5 vs 19.0 U, p = 0.04; Figure 4). Receiver-operator characteristic analysis demonstrated that hMR had a numerically superior diagnostic accuracy over IMR to predict the presence of extensive MVO (superior sensitivity), but this was not significant (AUC 0.83 vs 0.72, p = 0.22) (Figure 6). A threshold of  ≥ 3.25 mm Hg·cm^-1^·s provided the highest sensitivity (0.78) and specificity (0.74) for detecting extensive MVO. The best cutoff for IMR was ≥40 U with sensitivity (0.44) and specificity (0.91). In addition, hMR had superior diagnostic accuracy over IMR to predict the presence of any MVO, but this difference was not significant (AUC 0.75 vs 0.66).

## Discussion

To our knowledge, this is the first study in humans to have simultaneously assessed the correlation of 2 invasive indices of MVR, Doppler-derived hMR and thermodilution-derived IMR, against each other and against independent measurements of MVD. The main findings of this study are (1) hMR and IMR correlate modestly with each other, and therefore cannot be considered equivalent predictors of MVD; (2) hMR had superior diagnostic accuracy over IMR to predict MVD determined invasively by CFR; (3) hMR had a clinically superior sensitivity over IMR to predict MVD determined by cardiac magnetic resonance-derived MPRI and extensive MVO, but there were no statistically significant differences observed; (4) an hMR threshold of ≥2.5 mm Hg·cm^-1^·s and an IMR threshold from 21.5 to 24 U were optimal for predicting MVD determined by CFR and MPRI; (5) in the infarct related artery after an AMI, an hMR threshold of ≥3.25 mm Hg·cm^-1^·s and an IMR threshold of ≥40 U were optimal for predicting MVD determined by extensive MVO.

Optimal assessment of MVD enables better risk stratification for adverse cardiovascular outcomes. In addition, in the setting of AMI after PPCI, instant MVR measurement could help select patients most likely to benefit from adjunctive pharmacotherapy (e.g., intracoronary GpIIbIIIa inhibitors^19^, ^20^). An accurate assessment of MVR can be performed safely in the cardiac catheter laboratory, across a broad spectrum of MVD in patients with AMI and stable angina, using either hMR or IMR. However, although equivalent hyperemic distal pressures were obtained from the 2 intracoronary guidewires, the overall correlation between hMR and IMR was far from strong (rho = 0.39). Therefore, discrepancies in the MVR measurements relate to differences in the estimation of flow. Each technique has inherent theoretical assumptions that are challenged in varying pathophysiologic states. Thermodilution-derived transit time is a surrogate of absolute coronary blood flow and is not indexed to the amount of myocardium subtended. Doppler flow velocity, however, decreases only by a fraction as branching occurs. Therefore, hMR may be less influenced by the amount of myocardium subtended than IMR.

Previous investigators have reported a wide range of prognostic thresholds for both hMR (2.5 to 3.6 mm Hg·cm^-1^·s^16^) and IMR (32 to 40 U^7^^,^^21^) in patients who have experienced a recent AMI. The thresholds we identified for hMR and IMR to predict the presence of MVO are similar to that previously reported.^8^, ^16^, ^22^ The thresholds for predicting MVD with CFR and MPRI, which are more sensitive measurements of MVD, are understandably lower for both hMR and IMR. Recently, Patel et al measured hMR and IMR directly after PPCI in 34 patients recruited with STEMI.^23^ Patel et al demonstrated that hMR had a superiority trend over IMR in predicting parameters of infarct size and impaired LV ejection fraction, but this failed to reach statistical significance.^23^ However, they did not include measurements of MVD in this comparison.

Several study limitations should be acknowledged. First, notwithstanding the detailed physiologic characterization of our study cohort, this is a study with a relatively small sample size. Second, there is currently no true reference standard measurement of microvascular function. In our study, we used multiple distinct methods of assessing microvascular function, which we believe represents the best available composite clinical surrogate for a true reference standard. CFR was chosen as the invasive measurement of MVD because it was readily obtainable in every patient and is utilized in clinical practice as a marker of MVD.^17^ Although a CFR threshold of <2.0 in unobstructed coronary arteries to predict MVD is somewhat controversial, hMR also performed better than IMR to predict CFR thresholds of <2.3 and <2.5 (used in previous studies to define MVD). Third, although we acknowledge that CFR can be affected by several hemodynamic factors and loading conditions, these conditions were minimized by ensuring that (1) baseline and peak measurements for Doppler and thermodilution were taken immediately after each other; (2) all hyperemic measurements were taken during steady-state hyperemia with intravenous adenosine; and (3) no other drugs or intravenous fluids were administered between Doppler and thermodilution measurements. Fourth, CMR late gadolinium enhancement was performed up to 6 days after AMI and therefore the measurements may be confounded by partial resolution of transient MVD after AMI. Nevertheless, this would be expected to affect both hMR and IMR to the same extent. Finally, it should be noted that there is no accepted dichotomous threshold for defining MPRI and MVO, and the values we have used may differ from some studies.

This prospective 2-center study assessed the correlation between Doppler-derived hyperemic MVR and thermodilution-derived IMR against each other and against independent reference measurements of MVD. We found that these 2 invasive indices are both predictors of MVD. However, only a modest correlation was found between hMR and IMR. Therefore, these measurements cannot be considered equivalent predictors of MVD.

## Supplementary Data

Appendix S1 Electronic supplementary material. Appendix S1
