# Supplementary material for: Doppler Versus Thermodilution-Derived Coronary Microvascular Resistance to Predict Coronary Microvascular Dysfunction in Patients With Acute Myocardial Infarction or Stable Angina Pectoris
Source: Am J Cardiol. 2018 Jan 1;121(1):1–8. doi: 10.1016/j.amjcard.2017.09.012 (PMC5746201; doi:10.1016/j.amjcard.2017.09.012)
Supplement: Appendix S1 — Electronic supplementary material. [file mmc1.docx]

**Electronic Supplementary Material**

**Methods**

In patients presenting with an acute ST-segment-elevation myocardial infarction (STEMI), verbal informed consent was sought in the cardiac catheterization laboratory followed by written informed consent within 24 hours of percutaneous coronary intervention, in line with other STEMI studies.^10^

A minimum of 3 T_mn_ measurements were acquired at rest and hyperemia, and T_mn_ measurements were repeated until the variability between the 3 T_mn_ values was minimal.^12^ Doppler data were sampled at 200 Hz. A polynomial filter was applied to refine the derivates of the velocity signal and then analysed offline with custom designed software (written in Matlab, Delphi v. 2010; Embarcadero, San Francisco, CA, USA). Three to six consecutive cardiac cycles were selected to obtain baseline and hyperemic APV. Doppler-derived hMR was calculated as P_d_ divided by APV at hyperemia (Figure 1A: mmHg·cm^-1^·sec),^1^ and thermodilution-derived IMR calculated as P_d_ divided by the inverse of the hyperemic average T_mn_ (Figure 1C: mmHg·seconds, or units [U]).^2^ Doppler-derived CFR was calculated by the ratio of APV at hyperemia and at baseline,^3^ and thermodilution-derived CFR was calculated by the ratio of mean T_mn_ at baseline and hyperemia.^4^ For the AMI patients, the TIMI flow grade ^5^ and corrected TIMI frame count^6^ were also calculated as previously described.

CMR high-resolution perfusion measurements were made in 3 LV short-axis slices using 0.075mmol/kg gadobutrol bolus injections (Gadovist®, Bayer Healthcare, Leverkusen, Germany). A saturation recovery gradient echo method was used (repetition time/echo time 2.7ms/0.9ms, flip angle 20°, 5x k-t BLAST acceleration, 11 interleaved training profiles, spatial resolution 1.3 x 1.3 x 8mm^3^, 90 dynamic images). The stress perfusion scan was performed during steady state hyperemia with peripheral intravenous adenosine

(140mcg/kg/min). The rest perfusion scan was performed 15 minutes later to allow CMR contrast redistribution. In AMI patients, late gadolinium enhancement images were obtained 15 minutes following 0.2 mmol/kg gadobutrol injection (Gadovist®, Bayer Healthcare, Leverkusen, Germany or Dotarem®, Guerbet, Villepinte, France). In patients who underwent perfusion-CMR the late gadolinium enhancement images were acquired 15 minutes following rest perfusion imaging. (In these patients a 0.05 mmol/kg gadobutrol top up injection was given immediately after the rest perfusion scan, so that 0.2 mmol/kg gadobutrol was given in total.)

Functional analysis of CMR data was performed offline by blinded investigators using dedicated software (CMR 42, Circle Cardiovascular Imaging, Calgary, Canada and QMassMR v7.5, Medis, Leiden, the Netherlands). Endo- and epicardial contours were drawn on cine short-axis images in systole and diastole to calculate volumetric LV ejection fraction, LV mass and regional wall motion assessment (via 16-segment American Heart Association (AHA) model segmentation). MPRI was derived from semi-quantitative perfusion analysis of the upslope of myocardial versus LV blood-pool signal intensity curves during the first pass of the CMR contrast agent through the myocardium as previously described (Online data supplement).^7^ MPRI was calculated the ratio of perfusion at hyperemia to that at rest, using a 16-segment AHA model. Quantification of late gadolinium enhancement to derive infarcted LV mass was performed as previously described.^8^ Briefly, infarcted tissue was defined as having a signal intensity > 5 standard deviations above remote normal myocardium (Figure 1D). MVO was manually delineated as an area of hypoenhancement within infarcted LV mass (Figure 1D).^9^ The MVO volume (in milliliters) was then quantified using the dedicated CMR software above. Extensive MVO was a pre-defined dichotomous variable when there was > 2mls MVO volume present.^10^

**Figure legends**

**Figure 7.** Quantitative analysis of cardiac magnetic resonance imaging perfusion scans.

1. 3-Tesla high-resolution perfusion, visually demonstrating an inferior perfusion defect.
2. Manual delineation of endocardial, epicardial and left ventricular (LV) blood pool contours, propagated throughout every image acquired in the scan.
3. Myocardial and blood pool signal intensity curves derived for 16 myocardial segments across 3 short-axis LV slices, using the American Heart Association (AHA) model.
4. Maximal upslope of myocardial signal intensity is calculated for all 16 AHA segments separately for the stress and rest perfusion scans.
5. Myocardial perfusion reserve index (MPRI) is calculated as stress divided by rest myocardial signal intensity. It is calculated for 16 AHA segments (calculated to 2 decimal point when highlighted). Reduced MPRI is shown in the inferior segments.

**
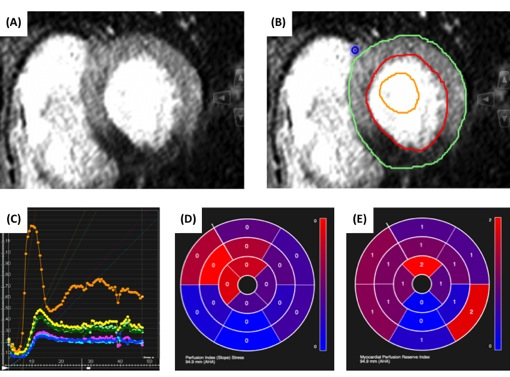
**

**References**

1. Meuwissen M, Chamuleau SA, Siebes M, Schotborgh CE, Koch KT, de Winter RJ, Bax M, de Jong A, Spaan JA, Piek JJ. Role of variability in microvascular resistance on fractional flow reserve and coronary blood flow velocity reserve in intermediate coronary lesions. *Circulation*. 2001;103:184-187.

2. Fearon WF, Balsam LB, Farouque HMO, Caffarelli AD, Robbins RC, Fitzgerald PJ, Yock PG, Yeung AC. Novel index for invasively assessing the coronary microcirculation. *Circulation*. 2003;107:3129-3132.

3. Marcus ML, Doty DB, Hiratzka LF, Wright CB, Eastham CL. Decreased coronary reserve: a mechanism for angina pectoris in patients with aortic stenosis and normal coronary arteries. *N Engl J Med*. 1982;307:1362-1366.

4. Pijls NH, De Bruyne B, Smith L, Aarnoudse W, Barbato E, Bartunek J, Bech GJ, Van De Vosse F. Coronary thermodilution to assess flow reserve: validation in humans. *Circulation*. 2002;105:2482-2486.

5. Chesebro JH, Knatterud G, Roberts R, Borer J, Cohen LS, Dalen J, Dodge HT, Francis CK, Hillis D, Ludbrook P, Markis JE, Mueller H, Passamani ER, Powers ER, Rao AK, Robertson T, Ross A, Ryan TJ, Sobel BE, Willerson J, Williams DO, Zaret BL, Braunwald E. Thrombolysis in Myocardial Infarction (TIMI) Trial, Phase I: A comparison between intravenous tissue plasminogen activator and intravenous streptokinase. Clinical findings through hospital discharge. *Circulation*. 1987;76:142-154.

6. Gibson CM, Cannon CP, Daley WL, Dodge JTJ, Alexander BJ, Marble SJ, McCabe CH, Raymond L, Fortin T, Poole WK, Braunwald E. TIMI frame count: a quantitative method of assessing coronary artery flow. *Circulation*. 1996;93:879-888.

7. Nagel E, Klein C, Paetsch I, Hettwer S, Schnackenburg B, Wegscheider K, Fleck E. Magnetic resonance perfusion measurements for the noninvasive detection of coronary artery disease. *Circulation*. 2003;108:432-437.

8. Kim RJ, Wu E, Rafael A, Chen EL, Parker MA, Simonetti O, Klocke FJ, Bonow RO, Judd RM. The use of contrast-enhanced magnetic resonance imaging to identify reversible myocardial dysfunction. *N Engl J Med*. 2000;343:1445-1453.

9. Wu KC, Zerhouni EA, Judd RM, Lugo-Olivieri CH, Barouch LA, Schulman SP, Blumenthal RS, Lima JA. Prognostic significance of microvascular obstruction by

magnetic resonance imaging in patients with acute myocardial infarction. *Circulation*. 1998;97:765-772.

10. Teunissen PF, de Waard GA, Hollander MR, Robbers LF, Danad I, Biesbroek PS, Amier RP, Echavarria-Pinto M, Quiros A, Broyd C, Heymans MW, Nijveldt R, Lammertsma AA, Raijmakers PG, Allaart CP, Lemkes JS, Appelman YE, Marques KM, Bronzwaer JG, Horrevoets AJ, van Rossum AC, Escaned J, Beek AM, Knaapen P, van Royen N. Doppler-derived intracoronary physiology indices predict the occurrence of microvascular injury and microvascular perfusion deficits after angiographically successful primary percutaneous coronary intervention. *Circ Cardiovasc Interv*. 2015;8:e001786.
